# Hydrogen-doped Brookite TiO_2_ Nanobullets Array as a Novel Photoanode for Efficient Solar Water Splitting

**DOI:** 10.1038/srep36099

**Published:** 2016-10-26

**Authors:** Mingi Choi, June Ho Lee, Youn Jeong Jang, Donghyung Kim, Jae Sung Lee, Hyun Myung Jang, Kijung Yong

**Affiliations:** 1 Department of Chemical Engineering, Pohang University of Science and Technology (POSTECH), Pohang 790-784, Korea; 2 Department of Materials Science and Engineering, and Division of Advanced Materials Science, Pohang University of Science and Technology (POSTECH), Pohang 790-784, Korea; 3 School of Energy and Chemical Engineering, Ulsan National Institute of Science and Technology (UNIST), Ulsan 689-798, South Korea

## Abstract

As a representative photocatalyst for photoelectrochemical solar water splitting, TiO_2_ has been intensively studied but most researches have focused on the rutile and anatsase phases because brookite, another important crystalline polymorph of TiO_2_, rarely exists in nature and is difficult to synthesize. In this work, hydrogen doped brookite (H:brookite) nanobullet arrays were synthesized via a well-designed solution reaction for the first time. H:brookite shows highly improved PEC properties with excellent stability, enhanced photocurrent, and significantly high Faradaic efficiency for overall solar water splitting. To support the experimental data, *ab initio* density functional theory calculations were also conducted. At the interstitial doping site that has minimum formation energy, the hydrogen atoms act as shallow donors and exist as H^+^. which has the minimum formation energy among three states of hydrogen (H^+^. H^0^, and H^−^). The calculated density of states of H:brookite shows a narrowed bandgap and an increased electron density compared to the pristine brookite. The combined experimental and theoretical results provide frameworks for the exploration of the PEC properties of doped brookite and extend our knowledge regarding the undiscovered properties of brookite of TiO_2_.

Over the past few decades, photoelectrochemical (PEC) hydrogen generation through solar water splitting has drawn increasing attention as a promising way of renewable energy production^1^,^2^,^3^,^4^. One of the crucial issues in the development of the ideal PEC cells having high solar-to-hydrogen conversion (STH) efficiencies is the synthesis of effective photoelectrodes. The ideal photoelectrode should satisfy several requirements, such as wide range of light absorption, fast charge separation/transport, high H_2_/O_2_ gas generation rate, long stability, and economic feasibility. Among various photoelectrode materials, a metal oxide semiconductor is considered as a strong candidate for an efficient photoelectrode^5^,^6^.

TiO_2_ is a representative stable metal oxide, which has been extensively investigated since the discovery of the photocatalytic property of TiO_2_ by Fujishima and Honda^1^. As a candidate for a suitable photoelectrode, TiO_2_ has unique merits, such as excellent stability, low cost, and nontoxicity; however, it also has a fatal demerit of having a large band gap energy of 3.2 eV (in the case of anatase), which restricts the utilization of solar energy. The utilization of visible light, which constitutes 45% of the total amount of solar energy, is essential to achieve a high STH efficiency. Several attempts have been made to utilize visible light with TiO_2_, e.g., quantum dot (QD) or dye sensitization, metal loading, and anion/metal ion doping of TiO_2_^7^,^8^,^9^,^10^. The development of heterostructures of TiO_2_ with a narrow band gap QD or dye, which can assist visible light absorption, has some obstacles, such as complicated fabrication processes that require several steps and instability of QD or dye (which can be decomposed and corroded in solution reactions). Another approach to utilize visible light is anion/metal doping of TiO_2_ to narrow the bandgap of the photoelectrode material. Since it was reported in 2001, doped TiO_2_ has been attracting great interest^9^,^10^,^11^,^12^.

TiO_2_ has three major crystalline polymorphs: rutile (tetragonal, space group: P42/mnm), anatase (tetragonal, space group: I41/amd), and brookite (orthorhombic, space group: Pbca). Most of the previous research studies of hydrogen treated TiO_2_ only focused on the anatase and rutile phases^13^,^14^,^15^; in contrast, brookite TiO_2_ has scarcely been studied because the brookite phase rarely exists in nature and is difficult to synthesize^16^. Although some research has been performed to discover the properties of doped brookite with calculated densities of states (DOSs), no single study has attempted to investigate the experimental analysis of properties of doped brookite due to the problem of the difficult synthesis^17^,^18^.

The aim of this research is to prepare and explore the undiscovered properties of hydrogen doped brookite (H:brookite), which can be fabricated using an innovate approach, and compare the experimental results with the theoretical calculations based on density functional theory (DFT). This work is the first study reporting the preparation and enhanced PEC overall solar water splitting properties of hydrogen doped brookite nanostructure. The high-quality single crystalline brookite nanostructures were prepared using a hydrothermal solution reaction and then doped with hydrogen gas using a high temperature furnace system. The morphologies and atomic structures of pristine and hydrogen doped brookite were compared, and doping of hydrogen in interstitial sites in brookite was confirmed by experimental and theoretical methods. H:brookite showed overall water splitting and enhanced PEC properties owing to narrowed bandgap and increased electron density which was analyzed experimentally and theoretically. The combined experimental and theoretical analysis results suggest that H:brookite TiO_2_ has a promising potential as a efficient photoanode for overall solar water splitting.

## Results and Discussion

### Morphology and crystal structure of brookite and H:brookite

**Figure 1:**
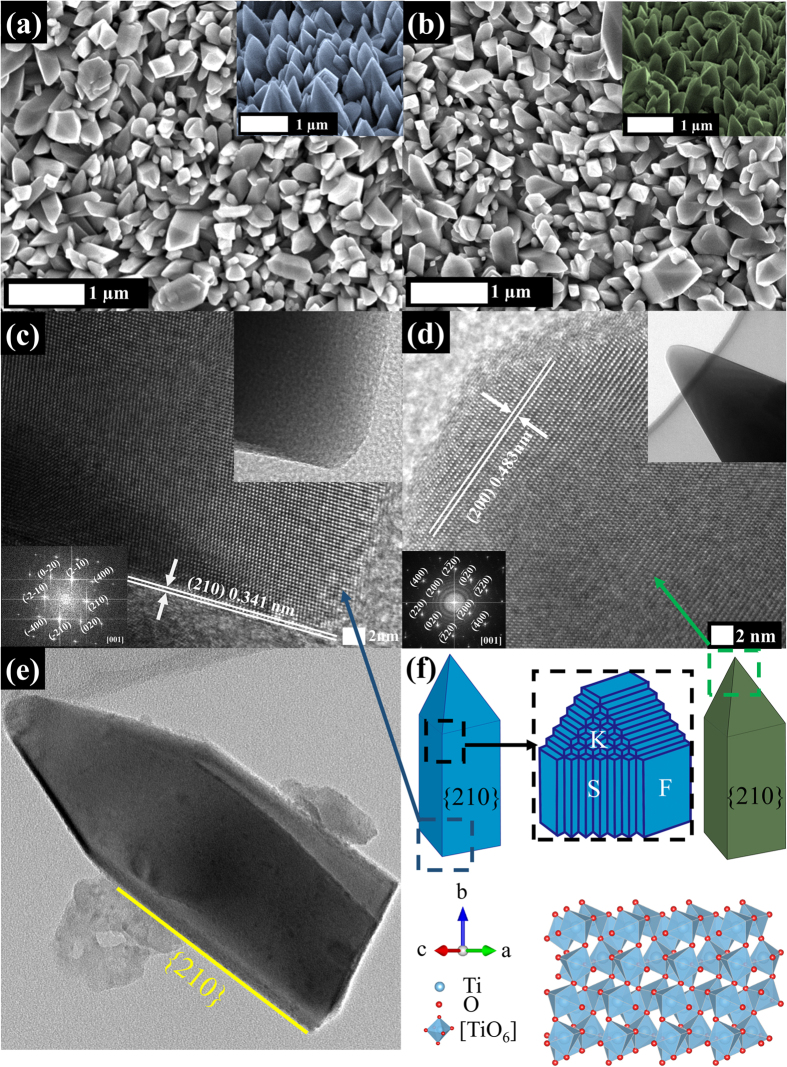
(**a,b**) Top-view SEM images of (**a**) pristine brookite and (**b**) H:brookite; the insets show the tilt-view SEM images. (**c,d**) HR-TEM images of (**c**) pristine brookite and (**d**) H:brookite; the insets show their FFT patterns and TEM images. (**e**) TEM image of the brookite bullet structure. (**f**) Scheme of bullet brookite structure and surface structures of {210} surface.

The brookite samples were synthesized using a well-designed solution reaction method^19^. The bullet shaped TiO_2_ nanostructures are vertically aligned on the titanium foil and thus do not require additional film deposition process for working electrode preparation, as is necessary for the powder materials. Figure 1 compares the morphology and crystal structure of brookite and H:brookite via field-emission scanning electron microscope (SEM) images. From Fig. 1a,b, hydrothermally grown TiO_2_ nanobullets are observed to have sharpened tips and are vertically aligned on the substrate (inset of tilt-view SEM images). In addition, the SEM analysis confirms that the morphology of the samples is not changed after annealing with a H_2_/N_2_ mixed gas for hydrogen doping.

The results obtained from the transmission electron microscope (TEM) analysis of the bottom part of brookite (green dashed rectangle in Fig. 1f) and the sharpened tip of H:brookite (red dashed rectangle in Fig. 1f) are presented the Fig. 1c,d, respectively. Both of their atomic structures were investigated using high-resolution TEM images that are recorded along the [001] zone axis, which show the high quality single-crystalline atomic arrangement. It can be seen from the left-bottom side insets in Fig. 1c,d that the fast-Fourier-transform (FFT) patterns corresponding to the HR-TEM images coincide with the diffraction patterns of brookite recorded along the [001] zone axis; thus, we believe that the crystal structures are not changed after annealing with a H_2_/N_2_ mixed gas. The distance between atomic planes, which are verified by the FFT patterns, were measured, indicating the d-spacings of the (210) and (200) atomic planes are 0.341 and 0.483 nm, respectively.

The unique bullet shape of the brookite TiO_2_ crystal is due to the exposed specific facets with low surface energies. Gibbs conclude that the crystals having the same volume should have a minimum surface energy at equilibrium^20^. Thus, when the crystals grow, they tend to adopt the shape that minimizes the total surface energy. Previous studies have reported that, according to their theoretical calculations, the most stable facet of brookite due to its low surface energy is {210}^21^,^22^. According to Curie’s theory regarding the formation of crystals, the growth rates of the crystal facets are proportional to the surface free energy^23^. As the reaction progresses, the crystal facets with fast growth rates disappear, and the most stable {210} facets with slow growth rates are extended to minimize the total surface energy. A kinetic roughening transition also occurs during the growth reaction at the crystal surface. In the case of metal oxide crystals, it is well known that the de-oxidation process ensues to preserve electronic neutrality, which can be deneutralized by adsorption of ions with different charges^23^. An illustration in the black dashed rectangle in Fig. 1f shows that, as a result of the roughening transition, stepped face (S) transform into a kinked face (K), which results in the sharp tip of crystal^23^. From Fig. 1c, we can see that lattice fringes of bottom edge are sharply enclosed. From the FFT, it can also be found that the sharply enclosed edges are {210} facets, supporting the explanation that brookite has dominant {210} facets by virtue of their low surface energy (Fig. 1e). Figure 1f shows the scheme of brookite structure and the structure of the exposed {210} surface, which is the most stable facet.

### SIMS and XPS analysis of brookite and H:brookite

**Figure 2:**
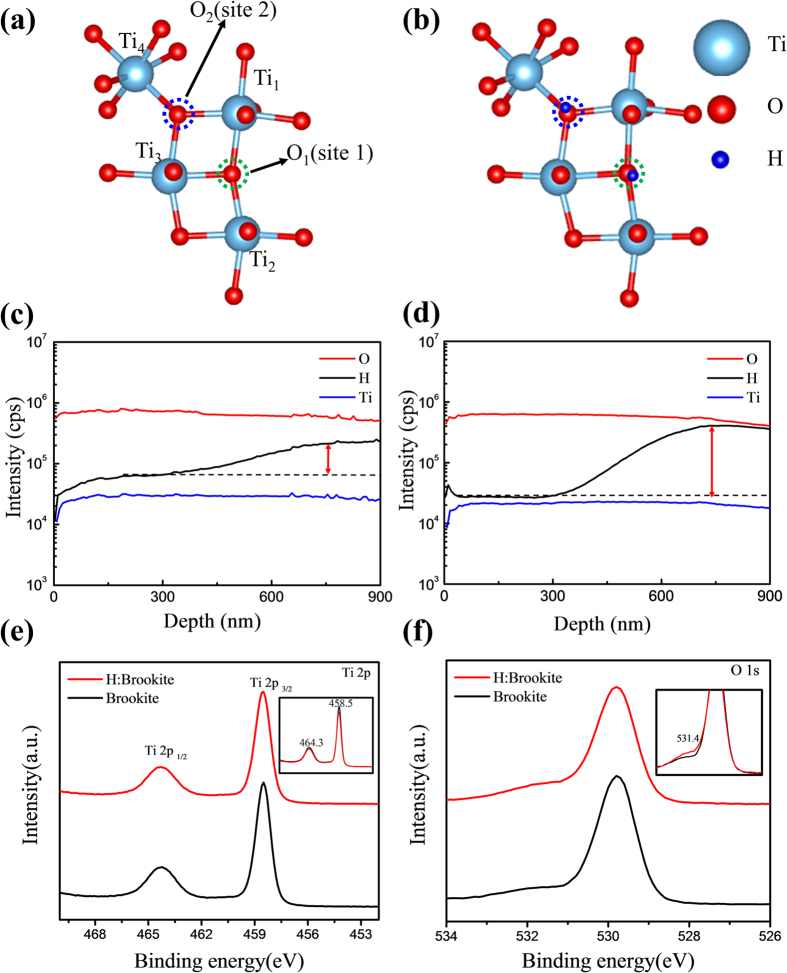
Crystal structure, which has two nonequivalent doping sites of (**a**) brookite and (**b**) interstitially hydrogen doped brookite. SIMS depth profile analysis of (**c**) brookite, and (**d**) H:brookite using a 15 keV Cs^+^ gun. (**e**) Ti 2p and (**f**) O 1 s XPS analysis of brookite and H:brookite using 15 kV monochromatic Al X-rays. The insets show their overlaid XPS results.

As shown in Fig. 2a, the brookite crystal structure has two nonequivalent doping sites. In the case of substitutional doping, shown in Figure S2d,e, hydrogen atoms will replace oxygen atoms, causing changes in the number of chemical bonds between titanium and oxygen atoms. However, as seen from Fig. 2b, interstitially doped hydrogen atoms will bond to oxygen atoms perpendicularly, which produces no changes in the number of chemical bonds between titanium and oxygen atoms^18^. Thus, there are four possible means for hydrogen doping, substitutional or interstitial doping on the two different sites, and all of their formation energies were calculated and compared in detail (see the theoretical analysis results presented in section *Formation Energy*). To verify the doping of brookite nanoarrays by experiments, we performed secondary ion mass spectrometry (SIMS) and X-ray photoelectron spectroscopy (XPS) analyses. SIMS is a widely used technique for determining the elemental distributions in a semiconductor (especially the dopant distribution), and it is applied to obtain depth profiles of elements in brookite and H:brookite. Figure 2c,d provide the SIMS depth profiles of brookite and H:brookite, respectively. The pristine brookite was immersed in an HCl solution during the fabrication process, which can provide the residual content of hydrogen in Fig. 2c. Contrary to the similar intensities of oxygen and titanium, the hydrogen ion intensity displays a remarkable increase in H:brookite samples compared to pristine brookite. From these results, we can surmise that H:brookite contains more hydrogen ion than pristine brookite.

We further investigated XPS experiments to analyze the chemical binding states of brookite and H:brookite. The Ti 2p and O 1s XPS spectra for brookite and H:brookite are presented in Fig. 2e,f, respectively. As shown in Fig. 2e and its inset, the Ti 2p XPS spectra of both samples are almost identical. Their Ti 2p_1/2_ and Ti 2p_3/2_ peaks occur at the binding energies of 458.5 and 464.3 eV, respectively, which correspond to the lattice chemical bonding of TiO_2_^24^,^25^. Their identical Ti 2p peaks indicate that the chemical bonding of titanium is not affected by heat treatment with hydrogen. On the contrary, Fig. 2f and its inset show a slight but noticeable change of the O 1s XPS spectra of the samples. The O 1s peak can be deconvoluted into two sub-peaks, which have binding energies of 529.8 and 531.4 eV, corresponding to the chemical bonding of TiO_2_ and OH, respectively^24^,^26^,^27^. It can be observed from Figure S1 that the deconvolution results of two samples show that the O 1s peaks appear at same peak positions, but have different ratios of the peak areas. The peak area ratio of OH to TiO_2_ of H:brookite is increased compared to that of pristine brookite. A possible explanation for these SIMS and XPS data might be that the hydrogen ions are doped into interstitial sites of the brookite after annealing with a H_2_/N_2_ mixed gas. If the hydrogen ions were doped into the substitutional sites, then the chemical bonding of brookite at Ti 2p should be changed^28^. However, according to our results in Fig. 2e, this change is not the case in our samples.

### Photoelectrochemical properties of brookite and H:brookite

**Figure 3:**
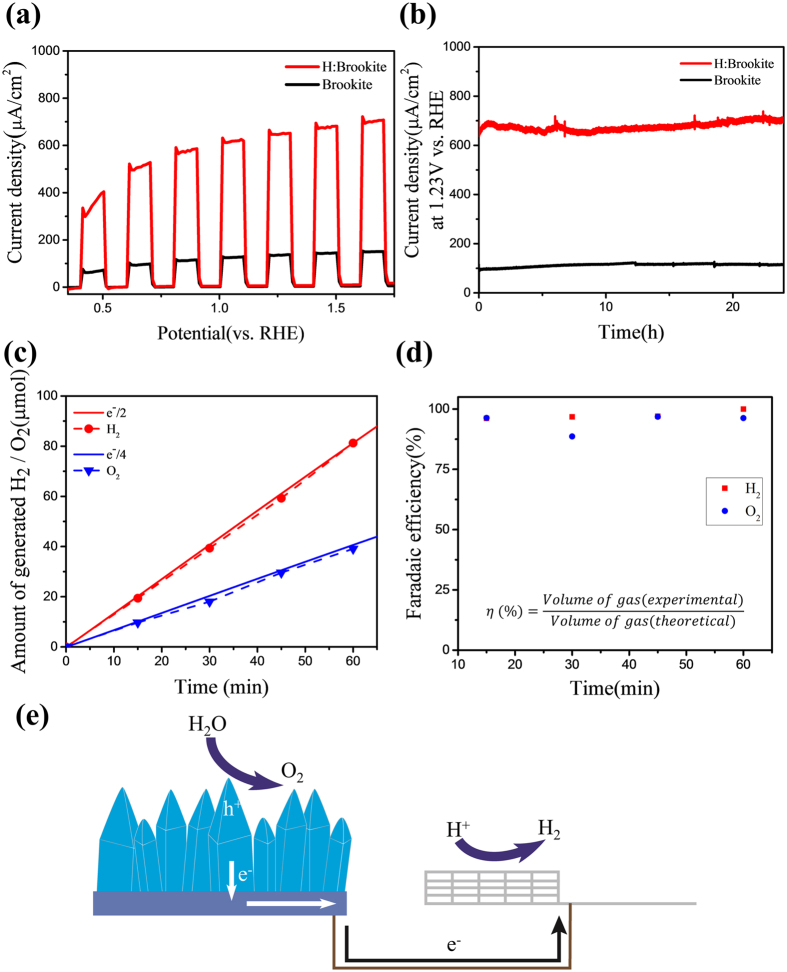
(**a**) Linear sweep voltammograms of pristine brookite and H:brookite electrodes prepared at 700 °C under chopped illumination (AM 1.5 G, 100 mWcm^−2^) (**b**) Chronoamperometry of H:brookite at 1.23 V vs. RHE under 1 sun simulated illumination (AM 1.5 G 100 mWcm^−2^) for 24 h (**c**) A comparison of the theoretical H_2_/O_2_ gas evolution calculated from photocurrent and the experimentally generated gas from the H:brookite photoanode. (**d**) The Faradaic efficiency equation and the calculated Faradaic efficiency for H_2_ and O_2_ gas evolution. (**e**) Schematic diagram of the water splitting process.

A heat treatment of brookite TiO_2_ with a H_2_/N_2_ mixed gas was conducted at various temperatures ranging from 500 to 800 °C. Photoelectrochemical (PEC) experiments were performed in a three-electrode electrochemical cell, with brookite or H:brookite, Pt flag, and Ag/AgCl as the working, counter, and reference electrodes, respectively, incorporating a 1 M NaOH electrolyte solution. The PEC characteristics of the cells were measured under a simulated air mass 1.5 G solar spectrum with intensity of 100 mWcm^−2^, which was adjusted using an NREL-certified silicon reference cell equipped with a KG-5 filter. The photocurrent density-voltage graphs under chopped illumination are shown in Fig. 3a. The 700 °C annealed sample has the best performance, whereas the heat treatment above 700 °C causes the sample to become exfoliated and wrecked. Thus, other experiments were performed on two samples: pristine brookite and brookite annealed at 700 °C with a H_2_/N_2_ mixed gas. When comparing pristine brookite and 700 °C annealed H:brookite, both samples show negligible dark currents, H:brookite shows an highly enhanced photocurrent density. In particular, the photocurrent at 1.23 V versus RHE increases more than five-fold from ~130 to ~650 μA after hydrogen doping.

As mentioned above, stability is one of the most important required characteristics of the photoelectrode for sustainable hydrogen generation. Figure 3b shows the stability test reults of brookite and H:brookite at 1.23 V versus RHE, indicating excellent stable performances over 24 hours. The discontinuous photocurrent of brookite at 12 h, and 18 h is attributable to the addition of electrolyte. In addition to measuring photocurrents, real gas generation was also measured by collecting hydrogen and oxygen gases. The produced gases from illuminated H:brookite at 1.23 V versus RHE were collected and analyzed by gas chromatography. The collected gases, which are identified as H_2_ and O_2_, versus time graph is presented at Fig. 3c. Water splitting properties of H:brookite are summarized in Table 1, showing amounts of H_2_ and O_2_ gas generations depending on collection times. The results indicate consistent ~2:1 volume ratio of hydrogen and oxygen, which corresponded to a theoretical volume ratio of H_2_:O_2_ upon overall water splitting. The theoretical volume of generated hydrogen and oxygen gas can be obtained by combining the Faraday’s law of electrolysis and the ideal gas law. The Faradaic efficiency (η) can be obtained by following equation^29^.


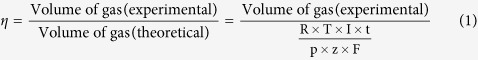


Where R, T, I, t, p, z, F denote ideal gas constant, temperature of the gas, current, time, pressure, number of electrons for releasing one molecule (2 for hydrogen gas, and 4 for oxygen gas), and the Faraday constant. The solid lines in Fig. 3c are theoretical H_2_/O_2_ gas evolution calculated from the equation (1), and they are almost coincident with the dashed lines, which are experimentally generated volume of gas. Figure 3d shows calculated Faradaic efficiencies, which exceed 95% except a single point, for water splitting. As shown in Fig. 3e, when H:brookite is illuminated, photo-generated electrons at H:brookite are transferred to Pt counter electrode via an external circuit and reduce hydrogen ions to generate hydrogen gases at the Pt flag, and photo-generated holes oxidize water to generate oxygen gases at surface of H:brookite. It is confirmed that photo-generated electrons and holes from illuminated H:brookite are fully utilized for overall water splitting with significantly high efficiency.

### UV-vis DRS and IPCE analysis of brookite and H:brookite

**Figure 4:**
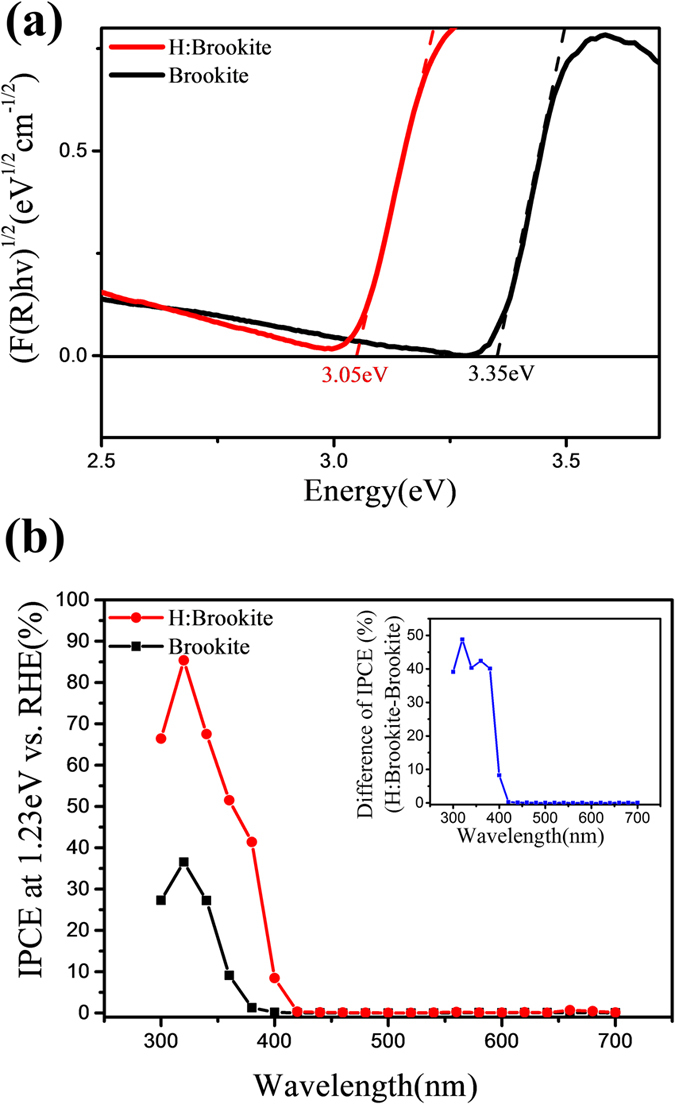
(**a**) Tauc plot of pristine brookite and H:brookite. (**b**) IPCE data of pristine brookite and H:brookite over the spectral range of 300 nm to 700 nm at intervals of 20 nm. Inset shows the difference of the IPCE value (H:brookite-brookite) as a function of the wavelength.

The light absorption properties of the samples were analyzed by UV-vis diffuse reflectance spectroscopy (DRS) with a 60-mm integrating sphere. Reflectance mode is applied in current study because our brookite nanostructures are grown on titanium foil and thus opaque. The bandgap of samples could be estimated by Kubelka-Munk function and the Tauc plot^2^,^30^,^31^. The Kubelka-Munk function, F(R), could be obtained from reflectance of the samples by the following equation,


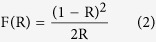


where R is the absolute reflectance of the sample^32^,^33^. The Tauc plot of the brookite, which is an indirect semiconductor, is described by the following equation,


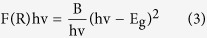


where *h* is the Plank’s constant, ν is the light frequency, B is the absorption constant for the indirect transition, and *E*_*g*_ is the optical bandgap^34^. In equation (3), the band gap could be estimated from the intercept of the tangent line and the energy axis of (F(R)hv)^1/2^ versus hv plot. As shown in the Fig. 4a, the bandgap of brookite and H:brookite is evaluated as 3.35 and 3.05 eV, respectively.

The narrowed bandgap of H:brookite is manifested by the incident photon-to-current efficiency (IPCE) results. The IPCE results indicate how efficiently the incident photon is converted to an electric current at a given wavelength. IPCE is the ratio of the number of output electrons to the number of input photons; thus, it can be calculated by following equation^35^,


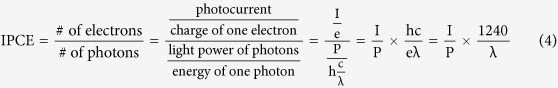


Where *I* is the measured photocurrent, *e* is the electron charge, *P* is the power of incident light, *h* is the Planck’s constant, *c* is the speed of light, and λ is the wavelength of incident light. The results obtained from the IPCE test of two samples are compared in Fig. 4b. H:brookite shows enhanced IPCE, especially for the incident photons of wavelength near 400 nm. This difference is due to the narrowed bandgap of H:brookite, which could absorb the light of longer wavelength range (up to 406 nm) than brookite (up to 370 nm). The difference of IPCE (H:brookite-brookite) depending on the wavelength in the inset of Fig. 4b supports the speculation that narrowed bandgap of H:brookite influences the IPCE. Contrary to the small difference of wavelength from 440 to 700 nm, a significant difference is found in the wavelength range from 380 to 400 nm that can only be absorbed by H:brookite. These results are consistent with the bandgap data obtained previously.

### Electrochemical impedance measurements of brookite and H:brookite

**Figure 5:**
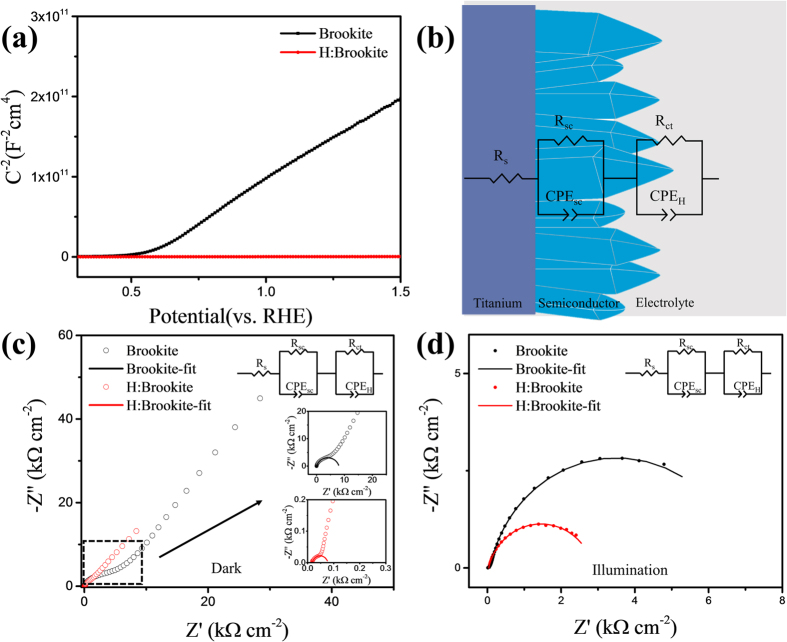
(**a**)Mott-Schottky plots of brookite and H:brookite. (**b**) Scheme of analogue electrical circuit of brookite(or H:brookite). Nyquist plots of EIS results of brookite and H:brookite measured under (**c**) dark and (**d**) 1 sun illumination condition at open circuit potential. Inset shows the enlarged high frequency region.

The Mott-Schottky plots obtained by the capacitance, which are determined by electrochemical impedance measurements, are shown in Fig. 5. From the Mott-Schottky plots, the carrier concentration of the samples can be calculated from reciprocal number of the slope by following equation^36^,^37^,


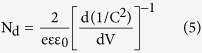


where *N*_*d*_, e = 1.602 × 10^−19^ C, ε = 64.1, ε_0_ = 8.854 × 10^−14^ F/cm, C, and V are the donor density, the electron charge, the dielectric constant of brookite, the vacuum permittivity, the capacitance, and the voltage, respectively^38^. In Fig. 5, the slope of the brookite graph is much greater than that of the H:brookite graph, which indicates that the electron density of H:bookite is greater than that of brookite. The obtained electron density with equation (5) of H:brookite and brookite is 1.07 × 10^22^/cm^3^ and 1.06 × 10^19^/cm^3^, respectively. A possible explanation for the increased electron density might be that the interstitially doped hydrogen atoms of H:brookite could act as an electron donor. Several studies have shown that interstitially or substitutionally doped hydrogen in metal oxides can act as a donor state^39^,^40^.

The scheme of electrical circuit having two RC(circuit with resistance and capacitor in parallel) elements is shown in Fig. 5b, where R_sc_ is resistance of semiconductor, CFE_sc_ is space charge capacitance, R_ct_ is semiconductor/electrolyte charge transfer resistance, CFE_H_ is Helmholtz capacitance^41^,^42^. The Nyquist plots of brookite and H:brookite obtained under dark condition at open circuit potential are presented in Fig. 5c. The semicircle at high frequency represents the charge transfer in the semiconductor, and the semicircle at low frequency is related to the charge transfer at the interface^41^. The arc at high frequency(the inset of Fig. 5c) of H:brookite is much smaller than that of brookite, implying that the H:brookite nanostructures exhibit lower R_sc_(80.3 Ω) than that of the brookite(6489 Ω), which is consistent with the result of Mott-Schottky plot. The semicircle related with charge transfer is too large to observe the whole of that, due to large resistance of semiconductor/electrolyte interface, which is consistent with the negligible dark current at Fig. 3a.

Figure 5d and Table S4 (presented in Supporting Information) provide the Nyquist plots and the parameters determined from EIS under 1 sun illumination condition at open circuit potential. It can be seen from the data in Table S4 that the resistance of overall charge transfer process under illumination is much lower than that of dark condition due to the photogenerated electrons. H:brookite has lower R_sc_(25.8 Ω) compared to the brookite(110 Ω) under illumination, due to hydrogen doping that results in increased electron density. The smaller arc at low frequency of H:brookite clearly indicates that the resistance of the charge transfer at the semiconductor/electrolyte interface of H:Brookite(6868 Ω) is much lower than that of brookite(2757 Ω). The enhanced charge transportation at the interface is attributed to the high conductivity of H:brookite, which facilitates more rapid transport of electrons to Pt electrode. Consequently, the residual holes don’t recombine with electrons, but transfer to electrolyte.

### Formation Energy

Density functional theory (DFT) calculations were performed to underpin the experimental results. Stabilized structure parameters of brookite have been calculated, revealing that the brookite possesses two distinguishable oxygen sites that are represented by different bond lengths and bond angles with nearby Ti atoms. Thus, hydrogen atoms could be interstitially or substitutionally doped at two different sites. Consequently, four distinct cases of hydrogen-doped brookite (after structural relaxation) have been calculated. The calculated structure parameters are described in the supporting information. When hydrogen atoms doped into the brookite structure, they would form a stable structure having the lowest formation energy, and it can be evaluated by using following equation:


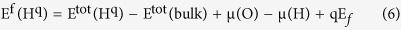


**Figure 6:**
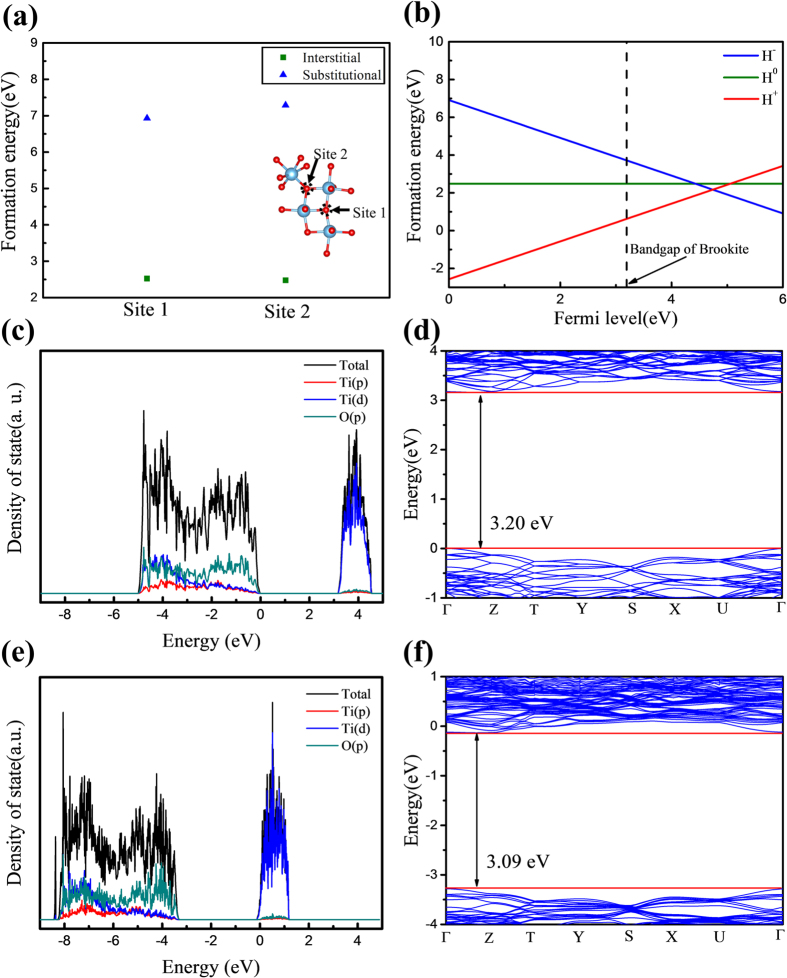
(**a**) The distinguishable two site of brookite structure and formation energies of hydrogen (H^0^) interstitial and substitutional doping at sites 1 and 2. (**b**) Formation energies of interstitially doped charged hydrogen (H^−^, H^0^, and H^+^) as a function of the Fermi level. Total density of states (TDOS) and partial density of states (PDOS) of (**c**) brookite and (**e**) interstitial H:brookite. Band structure of (**d**) brookite and (**f**) interstitial H:brookite.

Where *E*^*f*^(*H*^*q*^) is the formation energy of doped hydrogen atom, *E*^*tot*^(*H*^*q*^) is the total energy including doped hydrogen, *E*^*tot*^(*bulk*) is the total energy excluding doped hydrogen, *μ*(*0*) is the chemical potential of oxygen atom, *μ*(*H*) is the chemical potential of hydrogen atom, *q* is the charge and *E*_*f*_ is the Fermi energy. As shown in the equation (6), the formation energy depends on the charge state and the Fermi level. The computed formation energies at the zero Fermi energy of H^−^, H^0^ and H^+^ for the four possible doping states are compared in Table 2. Regarding the formation energies of H^0^, which is independent of the Fermi energy, the interstitial doping at the site 2 has the lowest formation energy. As shown in Fig. 6a, there is a significant difference in the formation energy between interstitial and substitutional doping. Thus, one can conclude that the hydrogen atoms will be doped at interstitial sites. At the interstitial site 2 which is the most stable doping site (Fig. 6a), the formation energy of H^+^ is much lower than those of H^0^ and H^−^ at the zero Fermi energy level (Fig. 6b). From this result, one can deduce the following scenario: The interstitially doped hydrogen atoms donate electrons and oxidize to H^+^. which has the lowest formation energy. As presented in Fig. 6b, the formation energy of charged states, such as H^−^ and H^+^. depends on the Fermi energy. Considering the band gap of brookite, which was measured as 3.35 eV, one can clearly conclude that H^+^ always has the lowest formation energy throughout the bandgap (Fig. 6b). Therefore, when hydrogen atoms are doped into brookite, they act as electron donors and oxidize to H^+^ states. This doping mechanism accords well with the experimental results of increased electron density as obtained by the Mott-Schottky plot.

### Density of states

The density of states (DOS) of both pristine and hydrogen-doped brookites were calculated using density functional theory to compare their electronic properties. Figures S3 and S4, respectively, present the DOS and the band structures of both pristine and hydrogen doped brookites with four feasible doping states. Figure 6c,e, respectively, show the DOS of brookite and interstitially doped brookite having the lowest formation energy. Two conclusions can be deduced from these computed results.

Firstly, the DOS of the H:brookite shows a higher Fermi level than pristine brookite, i.e., the conduction band minimum for the H:brookite (Fig. 6e) versus the valence band maximum for the pristine brookite (Fig. 6c). The higher Fermi level results from the increased electron density because the doped hydrogen acts as an electron donor at the doping sites. This increased Fermi level is consistent with the results obtained by the Mott-Schottky plot.

Secondly, the DOS of the H:brookite shows a narrowed bandgap. Figure 6d,f present the band structures of the pristine brookite and the interstitially doped brookite, respectively. From the band structures, we can find that the bandgap of the interstitially doped brookite is narrowed down, from 3.20 to 3.09 eV. The narrowed bandgap of the doped brookite results from the deformation of crystal structure in the course of the structural relaxation. The strong O-H bonds were formed by the interstitial doping; and after the relaxation, the configuration of crystal structures was changed. The changed bond length and angle (Table S1) caused by the doping consequently led to a narrower bandgap. On the contrary, as shown in Figures S3 and S4, the substitutionally doped brookite shows a high Fermi level and similar bandgap compared with brookite. Table 2 shows that H^+^ has the lowest formation energy when they doped into substitutional sites, thereby acting as an electron donor in the sites, and it leads to an increased electron density and a high Fermi level. From Table S3, one can find that the degree of structural variations by the substitutional doping is much smaller than that by the interstitial doping. The unchanged bandgap of the substitutionally doped brookite is due to a slight change in the crystal structure. These computed results reconfirm the interstitial doping of hydrogen in our brookite TiO_2_ samples.

The theoretical prediction of a narrowed bandgap by the H-doping correlates well with our IPCE and UV-vis experimental results, although the magnitudes of the bandgap are slightly different from each other. The small discrepancy can be attributed to the fact that *ab initio* DFT calculations correspond to 0 K (ground state) whereas the experimental results are obtained at room temperature.

## Conclusion

The present study is designed to determine the effect of hydrogen doping in the brookite via the combined analysis of experiments and DFT theory. Because brookite is difficult to synthesize, no single study has previously reported the properties of doped brookite. We successfully fabricated high-quality single crystalline brookite nanobullet arrays via a hydrothermal method and doped the brookite nanostructure with hydrogen for the first time. H:brookite shows excellent stability and enhanced photocurrent and can split water into hydrogen and oxygen gas with extremely high efficiency. We performed extensive analysis to elucidate doping effect. After hydrogen doping, the following were observed: (1) narrowed bandgap of H:brookite, as confirmed by DRS and IPCE tests, thus enabling utilization of the longer wavelength region of visible light and (2) increased electron density of H:brookite, as proved by the Mott-Schottky plot, due to increased number of electron donors caused by interstitially doped hydrogen. The *ab initio* prediction based on the DFT calculations further supports the experimental findings. The narrowed bandgap of the H:brookite was confirmed by the DOS and band-structure calculations. It has been shown that the increased electron density by the interstitial doping is directly correlated with the lowest formation energy of H^+^ and a high Fermi level (obtained from the DOS). The present experimental demonstrations and *ab initio* prediction of the water splitting properties of H-doped brookite provide us a framework for the exploration of undiscovered PEC properties of doped brookite and extends our knowledge to another important phase of TiO_2_.

## Experimental Procedures

### Materials

All the materials, such as titanium foil (foil, thickness of 0.25 mm, 99.7% trace metals basis), hydrochloric acid (HCl, ACS reagent, 37%), and sodium hydroxide (NaOH, pellets, semiconductor grade, 99.99% trace metals basis) were purchased from Sigma Aldrich.

### Fabrication of brookite nanostructures

First, the titanium foil (3 × 5 cm^2^), used as a substrate for TiO_2_ growth, was cleaned in a ultra-sonicator with water, acetone, and ethanol for 10 minutes. Next, the titanium foil was placed in a Teflon-lined stainless steel autoclave filled with 70 mL of 0.1 M NaOH aqueous solution. The autoclave reactor was placed in an electrical oven at 220 °C for 24 hours. After 24 hours, the reactor was cooled down at room temperature for 2 hours, and then the foil with TiO_2_ nanostructures was rinsed with water and ethanol. Afterwards, the foil was immersed in 1 M HCl solution for 10 minutes and then rinsed with water and ethanol. The foil was dried for 20 minutes at room temperature to remove moisture, and it was placed in a muffle furnace for heat treatment at 500 °C for 3 h at a ramping rate of 2 °C min^−1^.

### Fabrication of H:brookite nanostructures

After the heat treatment in a muffle furnace, the brookite nanostructures were obtained. The fabricated brookite nanostructures were annealed at 700 °C in a tubular furnace for 2 hours in a 4% H_2_/96% N_2_ mixed gas flow for hydrogen doping.

### Morphology and crystalline structure

The morphologies of the samples were observed using a field-emission scanning electron microscope (FE-SEM, XL30S, Philips) operated at a beam energy of 5.0 kV and high-resolution scanning transmission electron microscope (HR-STEM; JEM-2200FS with Image Cs-corrector; JEOL) operated at a beam energy of 200 kV. The crystalline structures were obtained via analysis of the fast-Fourier transformation (FFT) patterns of the HR-TEM images. The samples for TEM, which were exfoliated from brookite nanostructures and dispersed in ethanol, were deposited on a holey carbon grid and then were placed in an electrical oven for 1 day to remove the ethanol.

### Secondary Ion Mass Spectrometry (SIMS)

The experiments were performed in an UHV system for secondary ion mass spectrometry (SIMS, IMS 6f, Cameca) to obtain depth composition profiles. A Cs^+^ primary ion beam of impact energy of 15 keV was employed to analyze the samples. The beam current was 50 nA, and the beam reached the sample through a 150 μm × 150 μm size of raster; the generated secondary ions were collected by detector through 33π μm^2^ size of aperture.

### X-ray photoelectron spectroscopy (XPS)

X-ray photoelectron spectroscopy (XPS, ESCA LAB 250, VG scientific) data were taken by monochromatic Al-Kα radiation over an analysis area of 1.1 mm × 1.1 mm. The step size for survey scan was 1 eV, and the step size for the narrow scans performed for Ti 2p, O 1s, C 1s was 0.1 eV. The binding energy of the XPS spectra was calibrated with the reference to the C 1s peak at 284.4 eV.

### Photoelectrochemical cell properties

The photoelectrochemical properties of nanostructured brookite electrodes were measured using a typical three-electrode potentiostat system (potentiostat/galvanostat, Compactstat.e, Ivium Technologies) with a brookite nanostructure, and Ag/AgCl and Pt flag as the working, reference and counter electrode, respectively, incorporating a 1 M NaOH electrolyte solution, which was purged with N_2_ gas for 1 h. The working electrode was illuminated from the front side using a solar-simulated light source (AM 1.5 G filtered, 100 mW cm^−2^, Sun 3000 Solar Simulator, Abet Technologies).

### Electrochemical impedance measurements

Electrochemical impedance was measured in the same three-electrode system under dark and 1 sun illumination condition at open circuit potential, with the frequency range from 1 MHz to 100 mHz and the 10 mV of amplitude. The Mott-Schottky plots can be plotted by the voltage and capacitance which can be obtained with the three-electrode system, as mentioned above, in the dark via electrochemical impedance measurements at a frequency of 1000 Hz and 10 mV of amplitude.

## Additional Information

**How to cite this article**: Choi, M. *et al*. Hydrogen-doped Brookite TiO_2_ Nanobullets Array as a Novel Photoanode for Efficient Solar Water Splitting. *Sci. Rep.* **6**, 36099; doi: 10.1038/srep36099 (2016).

**Publisher’s note:** Springer Nature remains neutral with regard to jurisdictional claims in published maps and institutional affiliations.

## Supplementary Material

Supplementary Information

## Figures and Tables

**Table 1:** The water splitting properties of H:brookite versus the reaction time.

Time [min]	0	15	30	45	60
Amount of H_2_ gas [μmol]	0	19.4	39.4	59.3	81.3
Amount of O_2_ gas [μmol]	0	9.7	18.0	29.6	39.1
Ratio of H_2_ to O_2_		2.0	2.2	2.0	2.1
Faraday efficiency of H_2_ [%]		96	97	97	100
Faraday efficiency of O_2_ [%]		96	89	97	96

**Table 2:** Formation energy at the zero Fermi energy.

Location	E_f_ [eV]		
	H^0^	H^−^	H^+^
Interstitial (Site 1)	2.52	7.06	−2.53
Interstitial (Site 2)	2.48	6.91	−2.57
Substitutional (Site 1)	6.93	11.07	2.28
Substitutional (Site 2)	7.29	11.42	2.59
