# Supplementary material for: Hydrogen-doped Brookite TiO2 Nanobullets Array as a Novel Photoanode for Efficient Solar Water Splitting
Source: Sci Rep. 2016 Oct 26;6:36099. doi: 10.1038/srep36099 (PMC5080591; doi:10.1038/srep36099)
Supplement: Supplementary Information [file srep36099-s1.pdf]

Supplementary Information

Hydrogen-doped Brookite TiO_2 Nanobullets as a Novel Photoanode for Efficient Solar Water Splitting

Mingi Choi[1], June Ho Lee[2], Youn Jeong Jang[1], Donghyung Kim[1], Jae Sung Lee[3], Hyun Myung[2] Jang, and Kijung Yong[1]

[1]Department of Chemical Engineering, Pohang University of Science and Technology (POSTECH), Pohang 790-784, Korea

[2]Department of Materials Science and Engineering, and Division of Advanced Materials Science, Pohang University of Science and Technology (POSTECH), Pohang 790-784, Korea

[3]School of Energy and Chemical Engineering, Ulsan National Institute of Science and Technology (UNIST), Ulsan 689-798, South Korea

Correspondence: Professor Kijung Yong, Department of Chemical Engineering, Pohang University of Science and Technology (POSTECH), Pohang 790-784, Korea, E-mail: kyong@postech.ac.kr, Fax: +82-54-279-8298, Tel: +82-54-279-2278

S-I. XPS analysis of pristine Brookite and H:Brookite samples

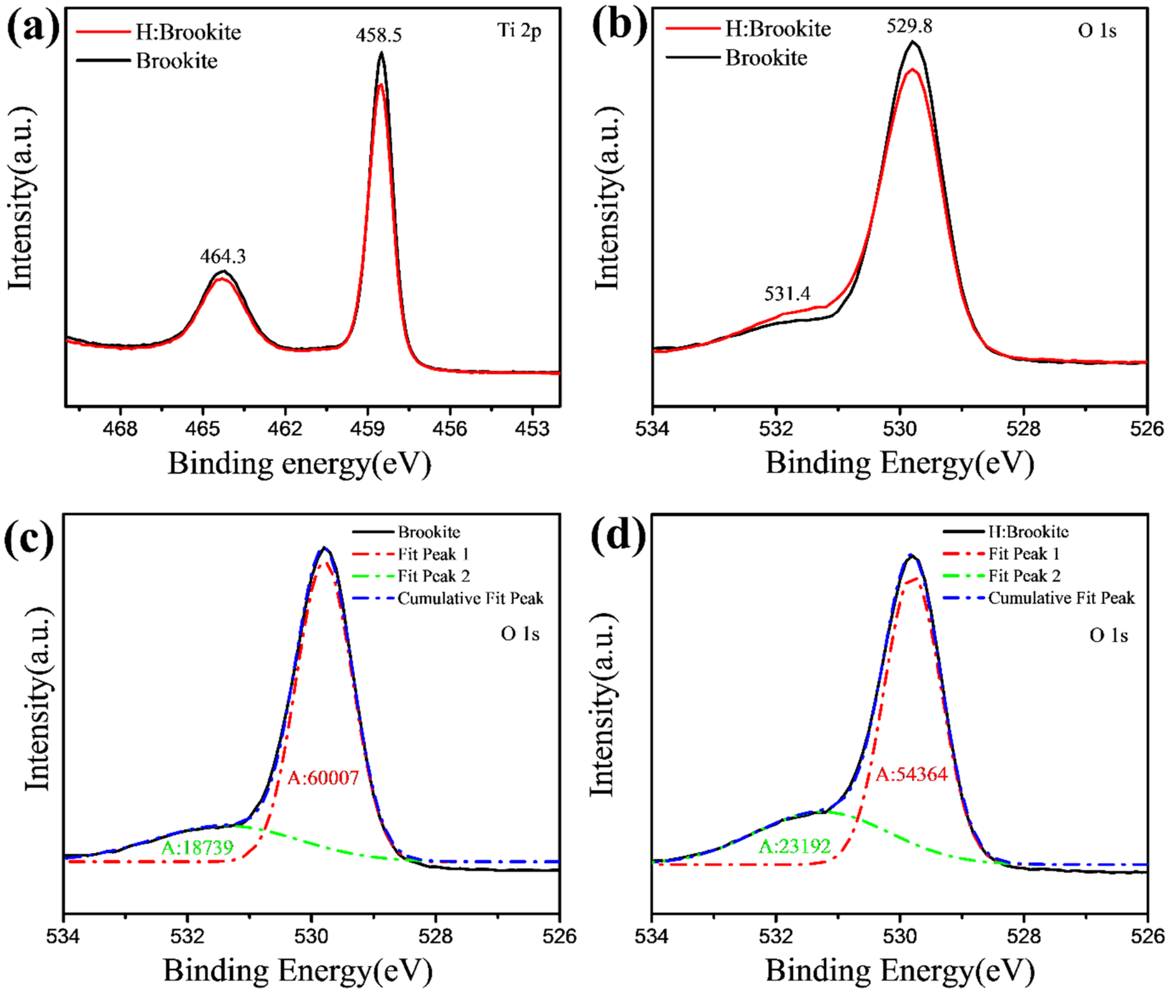

Figure S1. (a) Ti 2p and (b) O 1s overlaid XPS results of brookite and H:brookite using 15 kV monochromatic Al X-rays. Deconvoluted O 1s XPS peak of (c) brookite and (d) H:brookite.

S-II. Structure parameters

Theoretical calculations based on density functional theory were performed to support the experimental results. Stabilized structure parameters of brookite and four cases of hydrogen doped brookite after relaxation were calculated. As seen from Table S1 and Figure 2a, brookite had two distinguishable oxygen sites, which had different bond lengths and bond angles with nearby Ti atoms. Thus, hydrogen atoms could be interstitially or substitutionally doped at two different sites. In the case of interstitial doping, hydrogen atoms prefer to be located perpendicular to the Ti-O-Ti plane, as shown in Figure S3b, and S3c; this location is known to be stable.[1] Table S1 shows the structure parameters of two possible interstitial doping states. The strong O-H bonding of approximately 1Å due to interstitial doping of hydrogen causes the doped brookite structure to be stable. In the case of substitutional doping, hydrogen atoms replace the oxygen at sites 1 or 2; the crystal structure and structure parameters of substitutional doping states are presented in Figs. S3d and S3e and Table S2. The differences of bond length and angle are compared in Table S3. The bond lengths and angles of interstitial doped brookite were more greatly changed compared to those of substitutional doped brookite because of the strong O-H bonding of the interstitial doped brookite.

Table S1. Structure parameters of brookite and interstitially doped Brookite

**Bond length (nm)**	**Brookite**	**H:Brookite (Site 1)**	**H:Brookite (Site 2)**
Ti_1-O_1	1.96	2.07	1.95
Ti_2-O_1	1.94	2.04	1.96
Ti_3-O_1	1.99	2.17	1.99
Ti_1-O_2	1.96	1.95	2.08
Ti_2-O_2	1.99	2.01	2.11
Ti_3-O_2	1.96	1.96	2.07
H-O		0.99 (H-O_1)	1.01 (H-O_2)
**Bond angle(^{o})**	**Brookite**	**H:Brookite (Site 1)**	**H:Brookite (Site 2)**
Ti_1-O_1-Ti_2	162.3	165.1	158.0
Ti_2-O_1-Ti_3	97.5	93.8	96.6
Ti_3-O_1-Ti_1	99.1	95.2	103.9
Ti_4-O_2-Ti_1	131.8	129.2	129.2
Ti_1-O_2-Ti_3	99.3	104.6	95.2
Ti_3-O_2-Ti_4	125.0	122.8	120.6

Table S2. Structure parameters of brookite and substitutionally doped brookite

Bond length (nm)	Brookite	Bond length (nm)	H:Brookite (Site 1)	Bond length (nm)	H:Brookite (Site 2)
Ti_1-O_1	1.96	Ti_1-H_1	1.97	Ti_1-O_1	1.93
Ti_2-O_1	1.94	Ti_2-H_1	1.96	Ti_2-O_1	1.94
Ti_3-O_1	1.99	Ti_3-H_1	1.93	Ti_3-O_1	1.99
Ti_1-O_2	1.96	Ti_1-O_2	1.94	Ti_1-H_2	1.99
Ti_3-O_2	1.99	Ti_3-O_2	1.98	Ti_3-H_2	1.98
Ti_4-O_2	1.96	Ti_4-O_2	1.96	Ti_4-H_2	1.92

Bond angle (°)	Brookite	Angle (°)	H:Brookite (Site 1)	Angle (°)	H:Brookite (Site 2)
Ti_1-O_1-Ti_2	162.3	Ti_1-H_1-Ti_2	161.4	Ti_1-O_1-Ti_2	164.2
Ti_2-O_1-Ti_3	97.5	Ti_2-H_1-Ti_3	97.4	Ti_2-O_1-Ti_3	93.3
Ti_3-O_1-Ti_1	99.1	Ti_3-H_1-Ti_1	100.7	Ti_3-O_1-Ti_1	98.2
Ti_4-O_2-Ti_1	131.8	Ti_4-O_2-Ti_1	130.4	Ti_4-H_2-Ti_1	132.5
Ti_1-O_2-Ti_3	99.3	Ti_1-O_2-Ti_3	100.0	Ti_1-H_2-Ti_3	96.8
Ti_3-O_1-Ti_4	125.0	Ti_3-O_1-Ti_4	125.7	Ti_3-H_1-Ti_4	127.7

Table S3. Variation of the structure parameters of brookite after hydrogen doping

Bond length	Interstitial (Site 1)	Interstitial (Site 2)	Substitutional (Site 1)	Substitutional (Site 2)
Ti_1-O_1 (or H_1)	5.61%	0.51%	0.51%	1.53%
Ti_2-O_1 (or H_1)	5.15%	1.03%	1.03%	0%
Ti_3-O_1 (or H_1)	9.05%	0%	3.01%	0%
Ti_1-O_2 (or H_2)	0.51%	6.12%	1.02%	1.53%
Ti_2-O_2 (or H_2)	1.00%	6.03%	0.50%	0.50%
Ti_3-O_2 (or H_2)	0%	5.61%	0%	2.04%
**Bond angle**	**Interstitial (Site 1)**	**Interstitial (Site 2)**	**Substitutional (Site 1)**	**Substitutional (Site 2)**
Ti_1-O_1 (or H_1)-Ti_2	1.73%	2.65%	0.55%	1.17%
Ti_2-O_1 (or H_1)-Ti_3	3.79%	0.93%	0.10%	4.31%
Ti_3-O_1 (or H_1)-Ti_1	3.94%	4.84%	1.61%	0.91%
Ti_4-O_2 (or H_2)-Ti_1	1.97%	1.97%	1.06%	0.53%
Ti_1-O_2 (or H_2)-Ti_3	5.34%	4.13%	0.70%	2.52%
Ti_3-O_2 (or H_2)-Ti_4	1.76%	3.52%	0.56%	2.16%

S-III. Crystal structures of pristine, interstitially, and substitutionally doped Brookite

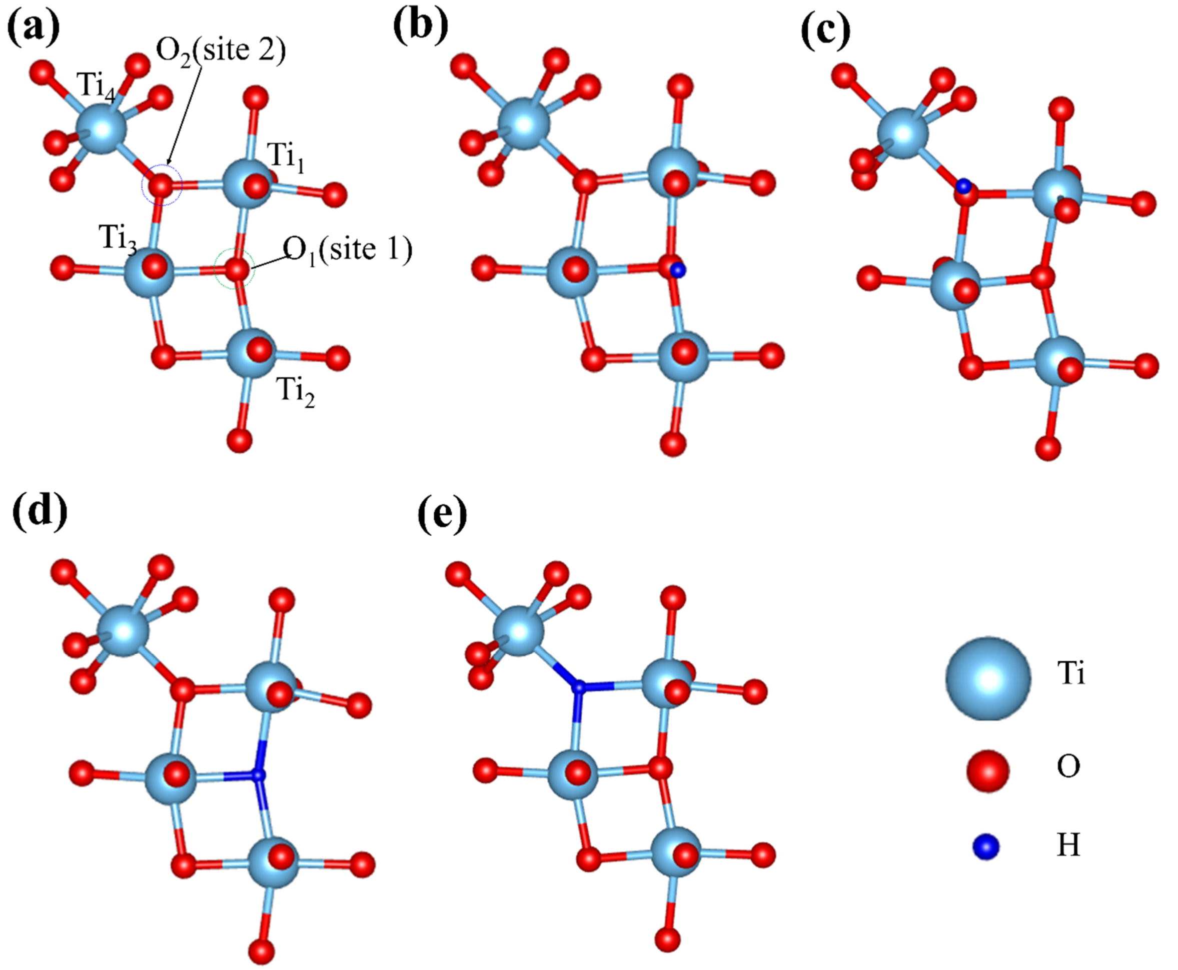

Figure S2. Crystal structure of (a) brookite, (b) interstitially doped brookite at site 1, (c) interstitially doped brookite at site 2, (d) substitutionally doped brookite at site 1, and (e) substitutionally doped brookite at site 2.

S-IV. Density of states of pristine, interstitially, and substitutionally doped brookites

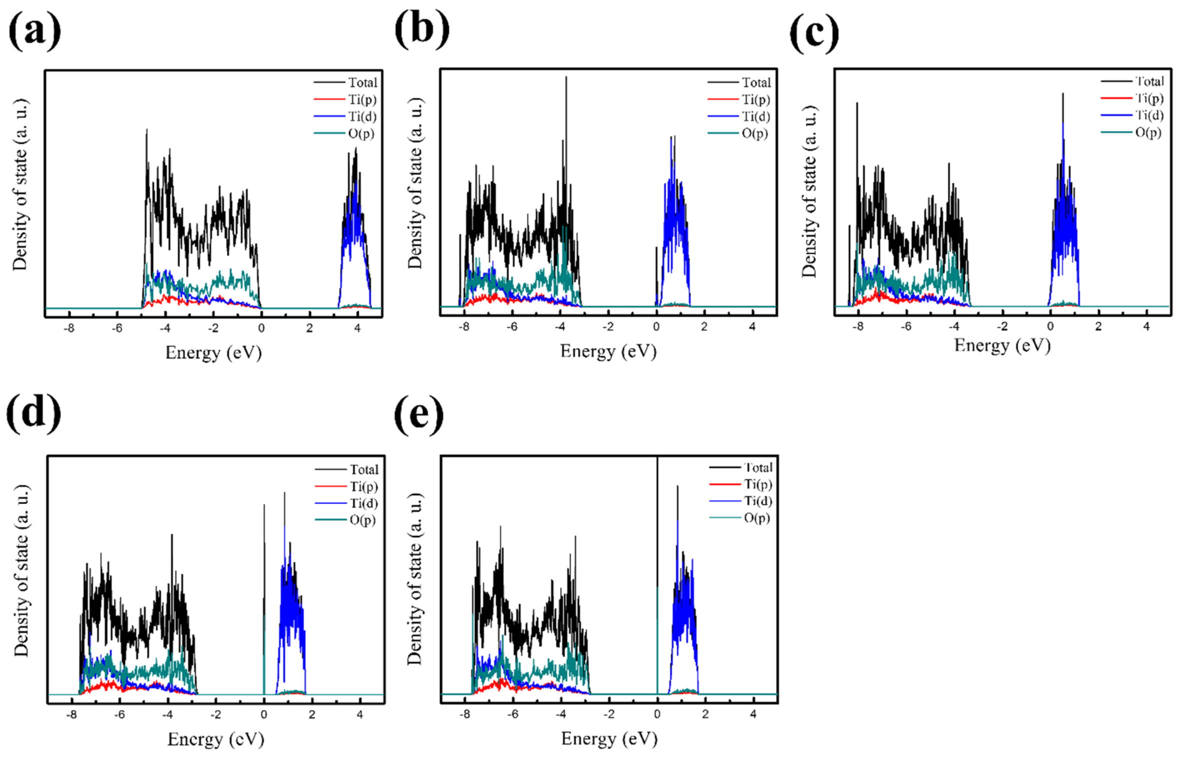

Figure S3. Total and partial density of states of (a) brookite, (b) interstitially doped brookite at site 1, (c) interstitially doped brookite at site 2, (d) substitutionally doped brookite at site 1, and (e) substitutionally doped brookite at site 2.

S-V. Band structures of pristine, interstitially, and substitutionally doped brookites

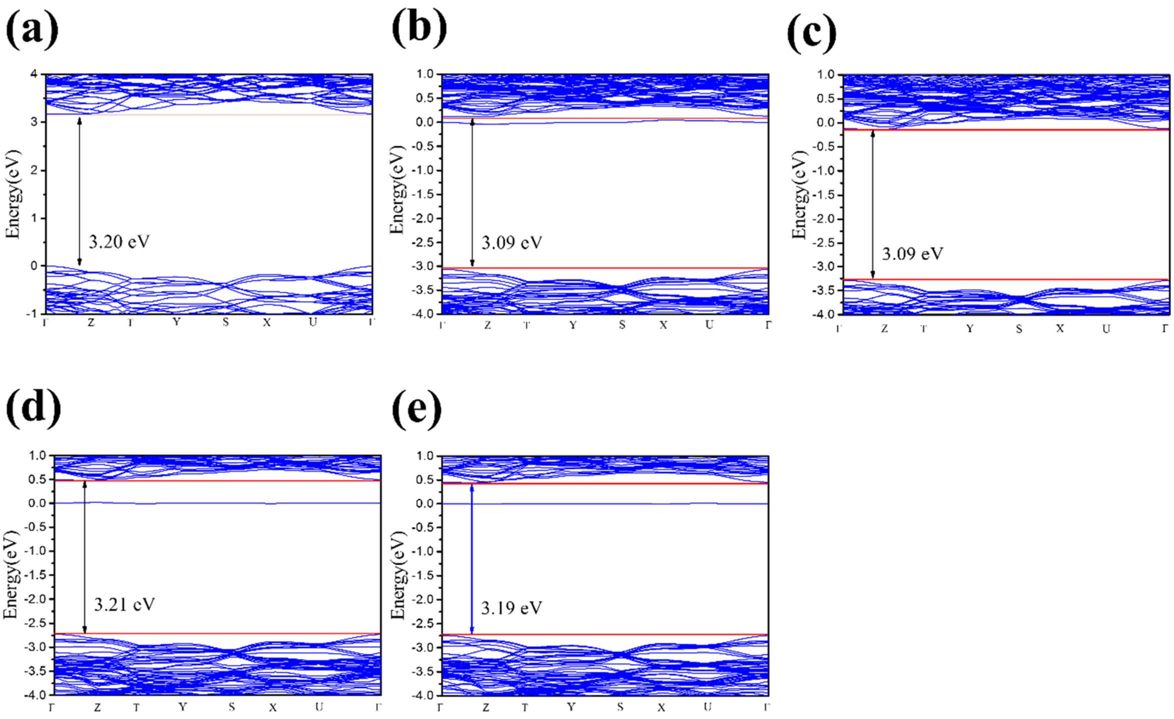

Figure S4. Band structures of (a) brookite, (b) interstitially doped brookite at site 1, (c) interstitially doped brookite at site 2, (d) substitutionally doped brookite at site 1, and (e) substitutionally doped brookite at site 2.

S-VI. Analysis of EIS data: parameters of brookite and H:brookite determined by fitting the electrical circuit model.

Table S4. Parameters of brookite and H:brookite determined by fitting the electrical circuit model to EIS data.

Sample	R_s [Ω]	R_{sc} [Ω]	CPE_{sc} [μF]	R_{ct} [Ω]	CPE_H [μF]
Brookite	11.5	110.04	364.37	6868	91.31
H:Brookite	19.11	25.84	371.68	2757	89.96

S-VII. Crystal structure and I-V curve of nitrogen doped brookite.

For nitrogen doping of TiO_2, the fabricated brookite nanostructures were annealed at 700 °C in a tubular furnace for 2 hours in a 67% NH_3/33% Ar mixed gas flow. However nitrogen doped brookite (N:brookite) has poor photoresponse which is even lower than brookite. From the DFT calculation results of nitrogen doped brookite, nitrogen and oxygen make strong chemical bonding, which weaken Ti-O chemical bonding of the structure. As a result, nitrogen doped brookite has unstable structure, which may explain the degraded PEC performances of nitrogen doped samples.

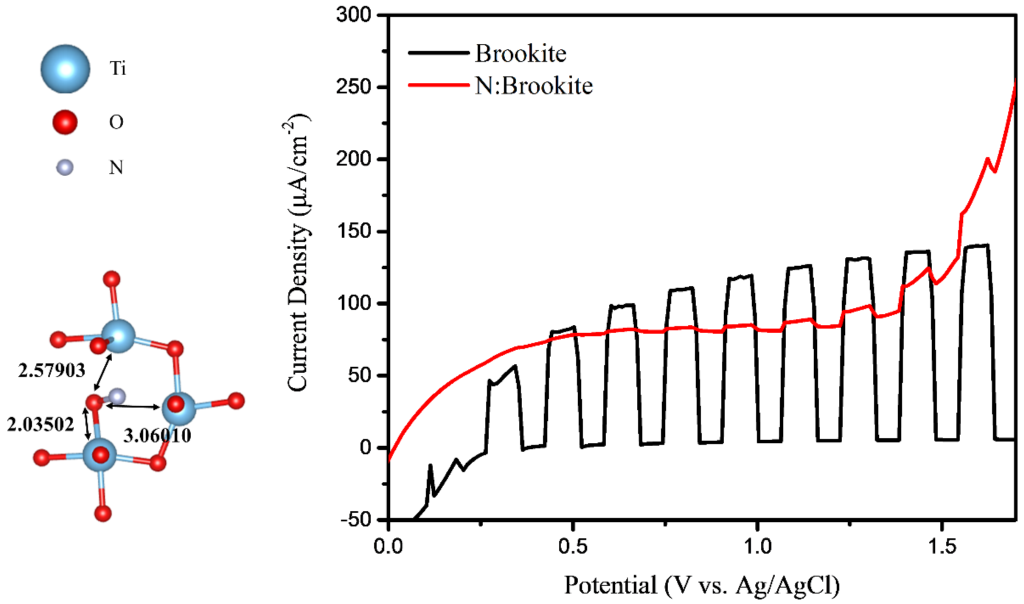

Figure S5. Crystal structure of nitrogen doped brookite and the linear sweep voltammograms of pristine brookite and N:brookite electrodes prepared at 700°C under chopped illumination (AM 1.5G, 100 $mWcm^{-2}$).

1. Pan, H., Zhang, Y.-W., Shenoy, V. B. & Gao, H. Effects of H-, N-, and (H, N)-doping on the photocatalytic activity of TiO2. *J. Phys. Chem. C* **115,** 12224–12231 (2011).
